# Outcome after Modified Maquet Procedure in dogs with unilateral cranial cruciate ligament rupture: Evaluation of recovery limb function by use of force plate gait analysis

**DOI:** 10.1371/journal.pone.0256011

**Published:** 2021-08-11

**Authors:** Giovanni Della Valle, Chiara Caterino, Federica Aragosa, Fabiana Micieli, Dario Costanza, Cristina Di Palma, Alfonso Piscitelli, Gerardo Fatone

**Affiliations:** 1 Department of Veterinary Medicine and Animal Production, University of Naples “Federico II”, Naples, Italy; 2 Interdepartmental Center of Veterinary Radiology, University of Naples “Federico II”, Naples, Italy; 3 Department of Agricultural Sciences, University of Naples “Federico II”, Portici, Italy; University of Bologna, ITALY

## Abstract

**Introduction:**

Cranial cruciate ligament failure is one of the principal causes of canine lameness. Several surgical procedures were proposed to achieve joint stabilisation; among these, the Modified Maquet Procedure involves using a titanium foam wedge to achieve and maintain the tibial tuberosity advancement. The force-plate analysis provides to objectively assess normal and abnormal gait and the outcome of different surgical techniques. The study evaluates the outcome of limbs that underwent Modified Maquet Procedure using land-force plate analysis comparing the operated limb with its healthy contralateral one as a control.

**Materials and methods:**

Thirty-five dogs with unilateral cranial cruciate ligament rupture were evaluated. Outcome after surgery was assessed by orthopaedic and radiographic evaluations and force plate gait analysis performed before surgery, at 15, 30 and 90 days after surgery. For objective comparison of ground reaction forces, data of operated limb were compared to contralateral limb on each time control and Symmetry Index at 90 days was determined. Healing radiographic signs, *minor* and *major* complication were reported.

**Results:**

A significant improvement in ground reaction forces was reached in all the treated limbs between set time intervals. The median percentage increase in ground reaction forces was constant from 15 to 90 days, with a Symmetry Index >9 in 54.2% of patients suggesting a normal gait symmetry. A complete bone healing was noticed at 90 days follow-up radiographic assessment. We experienced three *major* (8.5%) and one *minor* (2.8%) complications.

**Conclusions:**

To the Authors’ knowledge, this is the first study in the veterinary literature that assessed outcomes of dogs undergoing Modified Maquet Procedure for the treatment of cranial cruciate ligament rupture using force plate gait analysis and healthy contralateral limb as a control group. Our results confirm that Modified Maquet Procedure is an effective method to stabilise the stifle joint.

## Introduction

Several surgical techniques have been described in the veterinary literature to address the cranial cruciate ligament (CCLR) rupture considered the most common causes of pelvic limb lameness in dogs [1]. Proximal tibial osteotomies such as tibial plateau levelling osteotomy (TPLO) and tibial tuberosity advancement (TTA) have become increasingly popular over the years [2, 3]. The use of TTA in dogs was firstly described in 2002 by Montavon et al. as a technique to neutralise cranial tibial thrust in dogs with CCLR [3]. Further adaptations of this technique include the Modified Maquet technique (MMT) and the Modified Maquet Procedure (MMP, Orthomed, Huddersfield, UK) [4–6]. The latter technique was described in 2014 by Ness. It involves the placement of a titanium foam wedge (fixed in place by one pin and by cerclage wire or by a staple) to maintain the tuberosity’s advancement [5, 6].

The force-plate analysis is an objective, quantifiable and repeatable technique to assess normal and abnormal gait in dogs [7–11]. Measurement of ground reaction forces (GRFs) allows the examiner to describe gait symmetry and to evaluate limb loading; Peak Vertical Forces (PVFs) and Vertical Impulse (VI), in particular, when measured through the use of a pressure plate, were found to be reliable indicators of clinical lameness in dogs and have been therefore used as a method to assess clinical outcomes following surgical treatment for CCLR [7–14].

To our knowledge, there is only one study in the veterinary literature that assessed outcomes of dogs undergoing MMP for the treatment of CCLR using force plate gait analysis and healthy dogs as a control group [12].

This study aims to evaluate the outcome of limbs undergoing MMP surgery using land-force plate analysis at set time intervals and the contra-lateral healthy limb as a control.

## Materials and methods

### Animals

Medical records of dogs referred to “Federico II” Veterinary Teaching Hospital (Naples, Italy) for treatment of CCLR were reviewed. The presence of previous or concurrent orthopaedic and neurologic disease (such as lumbosacral disease, hip dysplasia, contra-lateral CCLR), weight inferior to 20 kg, and lack of complete force-plate data at every set time intervals were considered as exclusion criteria. Data recorded included dog signalment, surgical and radiographic findings, type of implant used, preoperative tibial plateau angle (TPA), pre-and postoperative patellar tendon-tibial plateau angles (PL-TPA) and any intra- and postoperative complication were recorded. Complications were classified as previously described by Cook et al. into *minor* (not requiring additional surgical or medical treatment to resolve) or *major* (requiring surgical or medical treatment to resolve) [15].

All procedures were performed for diagnostic and therapeutic purposes, and following the European directive 2010/63/EU, no ethical approval was needed for this study. Besides these procedures were carried out after informing the owners and getting their verbal consent.

### Preoperative evaluations

#### Clinical evaluation

A full orthopaedic and neurologic examinations were performed before surgery (T0) to assess for potential exclusion criteria and to confirm positivity/negativity of cranial draw and cranial tibial thrust tests and presence/absence of palpable joint effusion.

#### Force gait plate analysis

Force plate gait analysis was performed before surgery (T0) and at 15 (T1), 30 (T2), and 90 (T3) days post-surgery. A 40x40 cm platform (PASPORT Force Platform, PS-2141, PASCO scientific, California, USA) placed in a 4 m walkway was used to record GRFs.

Before data collection, dogs were let walking free across the walkway for at least 15 minutes to familiarise themselves with the environment and the operators. Each trial was considered valid when the pelvic limb and the thoracic limb fully struck at the same time the surface of the plate. The dogs were walked over the pressure plate until five valid trials were achieved. The dog’s velocity was registered with a dedicated detector (Motion Sensor II, CI-6742, PASCO scientific, California, USA), and only trials with a velocity of 1–1.3 m/s were accepted [16]. Dogs were walked in both directions with a standardised starting position.

Force-to-time curve was generated by the computer-analysis system (PASCO Capstone™ software 2.2.2, PASCO scientific, California, USA). Registered kinetic GFRs were collected for both pelvic limbs and included the peak of vertical force (PVF) and vertical impulse (VI). PVF was defined as the maximum force exerted perpendicular to the surface during the stance phase, while VI was the calculated area under the vertical force curve. Moreover, at T3-only, the pelvic limbs’ Symmetry Index (SI) was calculated to assess if lameness was still present [11]. As previously reported, an SI of 0 would indicate perfect gait symmetry whilst an SI > 9, at walking velocity, was considered indicative of persistent lameness [7]. As previously described, the GRFs parameters were normalised to body weight (PVF%BW, VI%BW) [17].

#### Preoperative radiographic assessment

A medio-lateral (ML) and a cranio-caudal (CrCd) radiographic view of the affected and healthy stifle was performed before surgery to confirm the presence/absence of joint effusion and to exclude concomitant stifle disease or femoral and tibial deformities. The ML view was performed with the joint positioned as closely as possible to 135 degrees and the femoral condyles over-imposed. Moreover, a ventrodorsal hip extended view (VDS) and a Ventrodorsal hip flexed and distracted view (VDD) [18] were acquired to rule out hip dysplasia and hip joint laxity. Finally, an ML view of the lumbo-sacral junction was obtained to exclude advanced lumbosacral disease.

The amount of required advancement of the tibial tuberosity was measured on the ML radiographic projection as previously described by Ness [6].

### Surgical technique

All dogs were premedicated with intramuscular (IM) administration of 0.02 mg/kg of acepromazine (Prequillan 10 mg mL ^-1^, Fatro Spa, Italy) and 0.3 mg/kg of methadone (Semfortan 10 mg mL ^-1^, Dechra Pharmaceuticals, UK). General anaesthesia was induced using intravenous (IV) administration of propofol (2–4 mg/kg; Proposure 10 mg mL ^-1^, Merial, France) and, after endotracheal intubation, maintained with isoflurane (Isoflo®, Abbott) in 100% oxygen. A nerve stimulated guided femoral and sciatic nerves block were performed using a total volume of 0.25 ml/kg of bupivacaine 0.25% (Bupivacaina Recordati; Recordati SpA, Italy) [19]. Intravenous cefazolin (22 mg/kg; *Cefazolina Teva*, *Italy*) was administered 30 minutes before the surgery and every 90 minutes until the end of the surgery.

The surgical technique was performed as previously described by Ness [5, 6]. The affected limb was clipped and aseptically prepared for surgery. A medial arthrotomy was performed and the use of a stifle distractor allowed inspection of the menisci and the cranial and caudal cruciate ligaments. Meniscal tears were treated by partial meniscectomy. The intact medial meniscus was treated by a medial meniscal release of the meniscal-tibial ligament. A dedicated saw guide (Orthomed, Huddersfield, UK) was used to perform the tibial tuberosity’s osteotomy. Progressive advancement of tibial tuberosity was obtained by inserting different temporary wedges (Orthomed, Huddersfield, UK) of increasing size until the desired advancement was obtained. The final titanium wedge was inserted in the osteotomy gap sitting 2 mm proximal to the Maquet hole. A 1.6 mm Kirschner wire (Alcyon, Italia) was inserted from cranial to caudal through the tibial tuberosity and the titanium wedge into the caudal cortex of the tibia. A 1.2 mm figure-of-eight cerclage wire or a dedicated staple (Orthomed, Huddersfield, UK) were used as a tension band. The soft tissue and skin were closed in a routine fashion. Immediate postoperative CrCd and ML radiographs (T1) were performed to evaluate the wedge’s correct position and the PL-TPA.

### Postoperative care

Methadone 0.1 mg/kg (Semfortan 10 mg mL^-1^, Dechra Pharmaceuticals, UK) was administered during hospitalisation for analgesia IV every 4 hours.

Firocoxib (5 mg/kg, once daily, for five days) and cephalexin (20 mg/kg, twice daily, for seven days) were dispensed at discharge.

Postoperative care consisted of 6 weeks confinement (cage or small room) when not on leash walking, which was allowed up to 6 times daily, beginning at 10 minutes duration per walk and gradually increasing to 30 minutes per walk by week 6. After six weeks, room confinement was allowed, but leash-only walking was encouraged for all dogs until week 12.

A follow-up orthopaedic examination was performed before force-plate analysis at T1, T2 and T3 to assess recovery progression and presence/absence of complications. Radiographic follow-up assessment was instead performed at T2 and T3 to document bone healing progression and exclude implant-related complications. Bone healing progression at the osteotomy site was considered satisfactory when new bone formation was noticed proximally and distally to the titanium wedge, and it was bridging the tibial tuberosity to the tibial diaphysis.

## Statistical analysis

The collected data were analysed using a specific statistics software package (IBM® SPSS® Statistics Version 26.0, IBM Corporation, Armonk, New York).

After verifying that the data of normalised kinetic variables (PVF%BW_T0_, PVF%BW_T1_, PVF%BW_T2_, PVF%BW_T3_, VI%BW_T0_, VI%BW_T1_, VI%BW_T2_ and VI%BW_T3_) were not normally distributed through Shapiro-Wilk tests, a 2-tailed Wilcoxon matched-pairs signed rank test was used to examine the differences of GFRs pre-and post-surgery (T0) and (T3) of the operated limb. The significance level for all variables between limbs and times was set a priori at *P* ≤ 0.05.

Besides, a 2-tailed Wilcoxon matched-pairs signed rank test was used to examine both the differences between healthy and affected limb values before surgery (T0) and the differences between normal and operated limb values 90 days after surgery (T3).

Finally, Friedman’s ANOVA test for related samples was used to assess if a statistically significant difference was noticed for each kinetic parameter of the operated limb over different time intervals. Pair-wise multiple comparisons, provided by Dunn-Bonferroni test, was used as a post hoc test.

## Results

Thirty-five dogs met the inclusion criteria of the study. The study population consisted of 20 females (8 neutered) and 15 intact males. At the time of presentation, mean ± standard deviation (SD) age and weight were respectively 66.3 ± 29.9 months and 36 ± 8.4 kg. Table 1 shows the distribution of canine breeds.

**Table 1:** Distribution of study population for breed, sex and wedge size.

Breed	Sex		Total	Wedge	
	Male	Female		Size	n
Labrador Retriever	6	5	11	9	5
				10.5	5
				13.5	1
Italian Cane Corso	2	1	3	10.5	2
				13.5	1
Pitbull Terrier	.	2	2	10.5	1
				12	1
Rottweiler	1	1	2	10.5	2
Golden Retriever	.	1	1	13.5	1
Dogue Argentine	1	.	1	10.5	1
Bull Mastiff	1	.	1	10.5	1
St. Bernard Dog	.	1	1	10.5	1
Mixed breed	4	9	13	9	5
				10.5	5
				12	1
				13.5	2
**TOTAL**	**15**	**20**	**35**		

**Fig 1:**
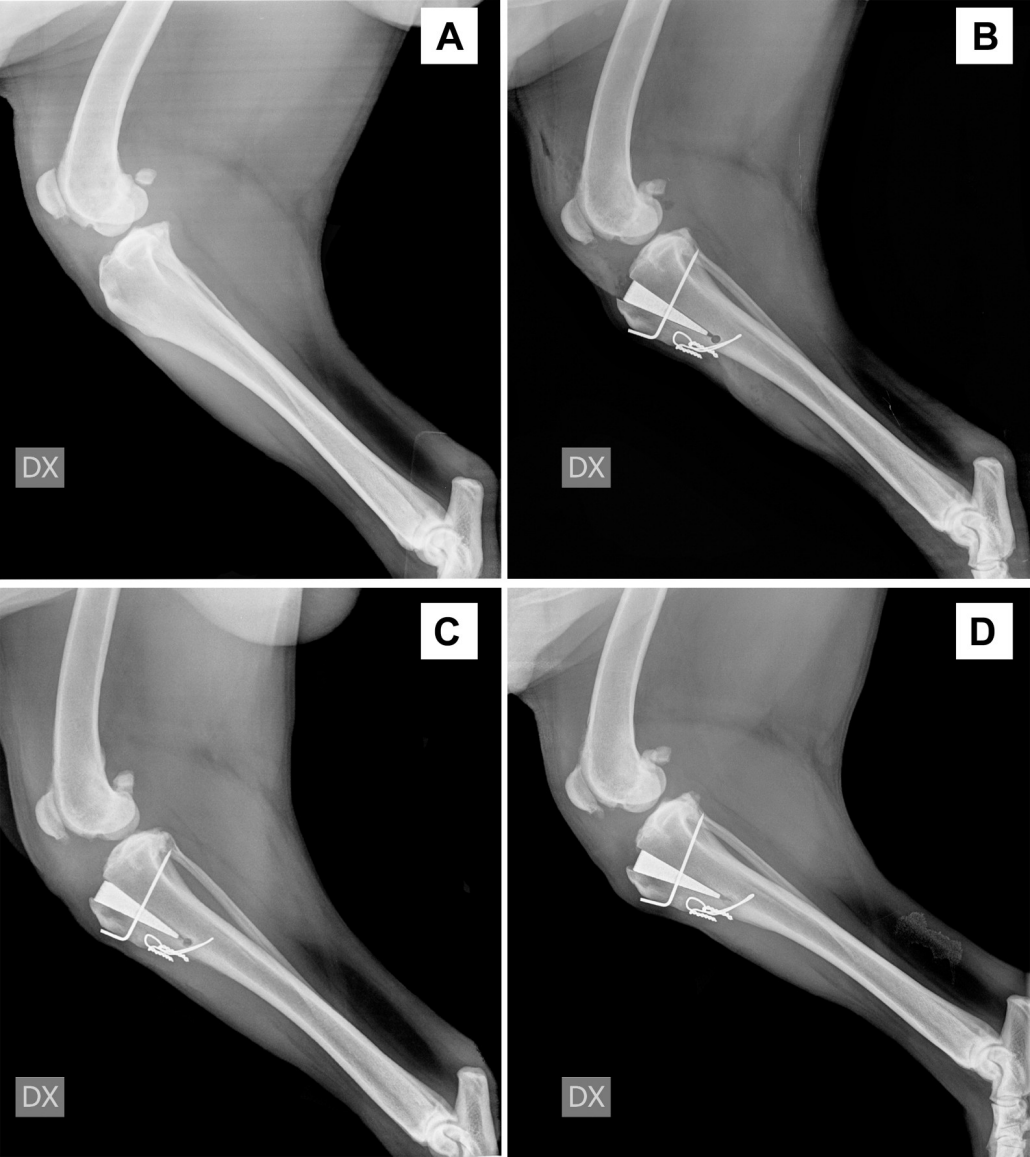
Radiographic evaluation over time intervals. Fig 1 A: preoperative radiograph (T0), B: immediate postoperative (T1), C: 30-days after surgery (T2), D: 90-days after surgery (T3).

In the study population, the menisci status appeared normal in 6 stifles (17.1%) while damaged in 27 (77.1%). The meniscal release was performed in 6 stifles (17.1%), and partial meniscectomy was performed in 27 stifles (77.1%). In two patients (5.7%), the meniscal status was not reported. Preoperative TPA was 24.3° (± 2°) (range 20° – 28°); pre-and postoperative PL-TPA were respectively 98.4° ± 4.3° (range 88°– 105.7°) and 89.7° ± 2.3° (range 87°– 95.6°). The sizes of the wedge used were 10.5 mm (17 stifles, 48.6%), 9 mm (10 stifles, 28.6%), 13.5 mm (6 stifles, 17.1%), 12 mm (2 stifles, 5.7%). The figure-of-eight cerclage wire was used in 34/35 patients (97.1%) (Fig 1); the staple was instead used in only 1/35 patients (2.8%).

**Fig 2:**
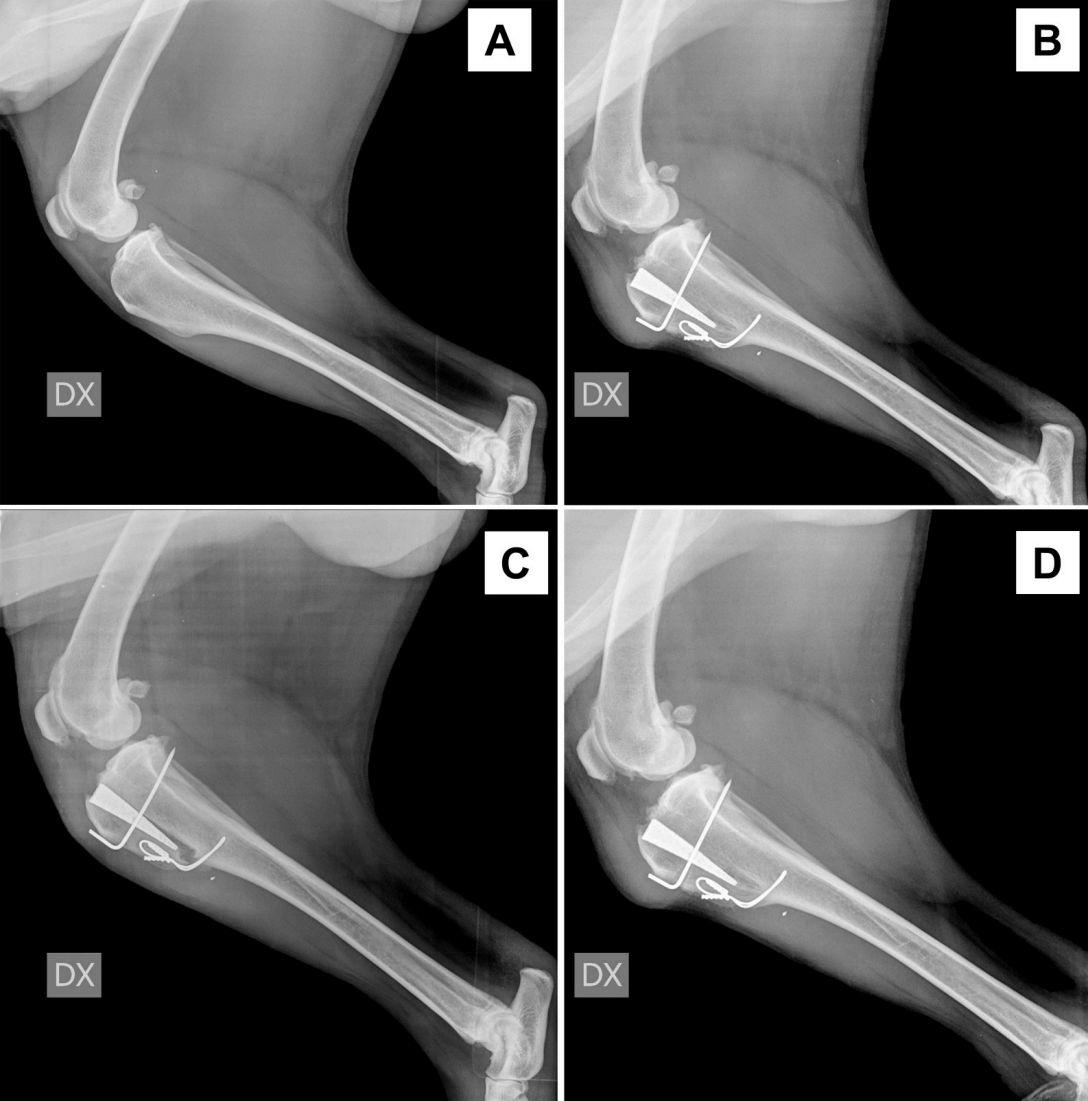
Radiographic evaluation of tibial tuberosity fracture over time intervals. Fig 2 A: preoperative radiograph (T0), B: immediate postoperative (T1), C: 30-days after surgery (T2), D: 90-days after surgery (T3).

No intra-operative complications were experienced. Immediate postoperative radiographs revealed a fissure of the cortical hinge propagating from the Maquet hole in 22 limbs (62.8%). None of these fissures received further surgical treatment, and all these dogs recovered uneventfully with complete bone healing noticed at T3 follow-up radiographic assessment. Three *major* (8.5%) and one *minor* (2.8%) complications were experienced in the postoperative period. Major complications included surgical site infection in one limb (treated with a course of cephalexin 20 mg/kg twice daily, for ten days) and a tibial tuberosity fracture in two limbs (Fig 2). The clients of the last two cases reported that a major trauma had occurred at 22 and 38 days post-surgery whilst the dogs were left unsupervised. The only *minor* complication that was encountered consisted of a seroma that spontaneously resolved within ten days.

Wilcoxon signed rank test on GFRs at T0 and T3 for the operated limb revealed a significant improvement of PVF%BW (p < 0.000) and VI%BW (p< 0.001). At T0, a statistically significant difference was present between GRFs of the healthy limb and the operated limb. (p< 0.000). After 90 days from surgery, despite there was an overall improvement in the kinetic parameters than before surgery, a statistically significant difference persists between healthy and operated limb for PVF%BW (p < 0.006) and VI%BW (p < 0.003).

As highlighted by Friedman’s ANOVA test results for related samples, significant differences in PVF%BW and VI%BW (p < 0.000) were shown over different time intervals on the operated limb (Table 2).

**Table 2:** GFRs for the operated limb from T0 to T3: Friedman’s ANOVA test values for related samples.

**PVF% BW**								
**T0**		**T1**		**T2**		**T3**		
**Median**	**Q1-Q3**	**Median**	**Q1-Q3**	**Median**	**Q1-Q3**	**Median**	**Q1-Q3**	***p-value***
28.52	[22.52–30.54]	27.97	[22.76–31.34]	31.01	[26.08–33.31]	34.78	[31.20–36.80]	0.000
**VI% BW**								
**T0**		**T1**		**T2**		**T3**		
**Median**	**Q1-Q3**	**Median**	**Q1-Q3**	**Median**	**Q1-Q3**	**Median**	**Q1-Q3**	***p-value***
10.06	[6.77–12.76]	9.88	[7.53–12.46]	12.04	[9.53–14.12]	13.64	[11.83–15.37]	0.000

The results of the pair-wise multiple comparisons (Dunn-Bonferroni test) indicate statistically significant differences between T0-T3 (p < 0.000) and T1-T3 (p < 0.001) for PVF%BW and between T0-T3 (p < 0.000) and T1-T3 (p < 0.000) for VI%BW of the operated limb.

Moreover, the median percentage increase in GFRs at each time point highlights a slight percentage decrease between T0 and T1 and a constant increase from T1 to T3 (Table 3).

**Table 3:** Median percentage increment of GFRs in operated limb from T0 to T3.

	T0-T1	T1-T2	T2-T3	T0-T3
**PVF% BW**	*-1*.*93%*	*10*.*87%*	*12*.*16%*	*21*,*95%*
**VI% BW**	*-1*.*79%*	*21*.*86%*	*13*.*62%*	*35*.*59%*

AT T3, the SI of 19/35 patients (54.3%) was < 9, suggesting that relatively normal gait symmetry was present. Residual lameness was instead noticed in 16/35 patients (45.7%) whose data showed a SI > 9.

## Discussion

This study described the successful improvement in limb function achieved by 35 dogs with unilateral CCLR that underwent MMP and whose outcome was objectively assessed by sequential force-plate analysis.

The MMP technique is, in our experience, a relatively straight-forward technique that, similarly to the TTA, allows the neutralisation of the shear forces of an insufficient stifle by achieving a final PL-PTA of 90° (± 5°). One of the main advantages of this technique compared to the traditional TTA and MMT consists in the use of a titanium foam wedge to support the advanced tibial tuberosity and to provide early osteointegration due to its conductive role scaffold [4–6]. A recent human study has demonstrated that bone tissue starts filling the pores of a titanium foam scaffold as early as four weeks after implantation [20]. Our sequential radiographs confirmed the titanium foam wedge’s early osteointegration at 30 days, followed by complete osteointegration by 90 days post-surgery. The reported complication rate following the MMP technique varies between 20% in a study reporting only a major complication rate [12], and 23% in a study in which minor and major complications rate are summarised [6]. The major complications reported in a recent study included late meniscal lesions, implant removal due to seroma formation, implant breakage or loosening, tibial fracture and wound complications [12]. Our study experienced a relatively low major complication rate (8.5%); one case developed a surgical site infection that required medical treatment to resolve, and two dogs suffered a tibial tuberosity fracture. One of these two cases required revision surgery with the application of a second figure-of-eight cerclage wire to stabilise the tuberosity 29 days after surgery. The second case showed a breakage of the pin, mild proximal displacement of the titanium wedge and the tibial tuberosity; this was managed conservatively with strict cage rest for an additional 4 weeks, and his condition improved within 2 months. Both patients underwent force gait plate analysis and radiographic evaluation at T3 and 150 days after surgery that showed complete fracture healing. Despite progressive amelioration of the GRFs, both these cases showed an SI > 9 that confirmed persistent lameness.

The minor complication rate that we experienced was low compared to the other clinical studies about MMP [6, 12]. We decided not to include the development of intra-operative fissure at the hinge cortex into the postoperative minor complication rate count, but if this is was to be considered, our minor complication rate could be as high as 65.7% [21]. One study reported that only 9.4% of these fissures subsequently led to postoperative fractures [22]. In our cases, we presume that the development of a fissure of the cranial hinge cortex had a relatively benign outcome because of the protective effect exerted by the cerclage wire tension band that was applied intra-operatively in almost all cases.

The thickness of the cortical hinge has been identified as a risk factor for the development of intraoperative fissures [23]. The speed at which the TTA is performed could also influence the development of these fissures, and gradual advancement of the tibial tuberosity over several minutes at small millimetres increments should reduce the incidence of fissure development [22].

Use of force-plate analysis provided accurate and repeatable data on limb function and an objective measurement of the efficacy of the MMP.

Trotting gait was shown to be more sensitive and accurate than the walking gait for the differentiation of dogs with low-grade pelvic limb lameness from normal ones using force-plate gait analysis [7]. However, a significant linear correlation was found between walk and trot in dogs with severe lameness [24]. Acquisition of valid trials can also be extremely challenging at the time of trotting a dog with severe lameness on the walkway [24], hence why, in the present study, we chose to acquire data at walk and not at trot.

Our data revealed an overall improvement of the GRFs at every set point except in the first 15 days immediately after surgery. A certain degree of discomfort caused by the surgical trauma is expected in the first few weeks after surgery, leading to a reduction in GRFs of the operated limb. However, at T2 and T3, a statistically significant improvement of GRFs was confirmed, suggesting that MMP seems to re-establish a relatively normal gait in the medium term. It is worthy to note that, 54.2% of cases were considered sound by SI analysis. However, a significant difference was not noticed for PVF and VI between healthy and operated limb at the T3 interval. These findings are in line with the results of a previous study that showed that TTA-treated cases did not achieve SI, similar to the normal control group until >300 days after surgery [25].

Moreover, at T3, in 12/35 patients (34%) for PVF and 10/35 patients (28%) for VI, respectively, the comparison between operated and normal limb showed an increased value of both GFRs for the operated limb. Even if, until the end stage of the study, no one bilateral CCLR was detected, these values could be interpreted as an initial failure of the contralateral cranial cruciate ligament. However, in our opinion, these data could reflect simply the weight shifting on the operated limb resulting more stable and painless of contralateral.

Force-plate analysis has been used in several studies to compare the clinical outcome of different surgical techniques objectively, and a cohort of healthy dogs has always been used as a control group [10–12, 14]. GRFs are influenced by conformation and body size, making a comparison with a control group not always reliable [26]. Previous study investigated MMP outcome using healthy Labrador Retriever as control group does not report a statistical comparison about, body size and conformation, and does not taking into account the morphometric variability into the breed [12].

To our knowledge, this is the first study assessing the clinical outcome of MMP by use of force-plate analysis and the contralateral healthy limb as a control.

There are several limitations of the present study that needs to be considered. The number of animals included in the study is low; this represents the difficulty in recruiting and evaluating only unilaterally affected patients. The short-term follow-up is due to the choice to assess the recovery limb function in the early postoperative period, and our cases may prove that dogs undergoing to MMP were significantly less lame in this critical period after surgery [7].

The use of two platforms rather than one (as used in our study) may have helped to minimise the variables involved with trial repetition [27]. Lastly, even if considered accurate for detection of residual lameness, use of SI in dogs where the contralateral limb is used as control could be influenced by several variables (e.g. dominant limb, behavioural lateralisation, paw preference and side shifting) [27].

The results of our study confirmed that MMP is an effective method to provide dynamic stabilisation in those stifle joints that suffer from cranial cruciate ligament insufficiency. The use of the contra-lateral healthy limb as a control during force-plate analysis allowed us to eliminate all those variables that would arise by using a different cohort of animals as a control group.

## References

[1] Vaughan LC. The history of canine cruciate ligament surgery from 1952–2005. Vet Comp Orthop Traumatol. 2010; 23: 379–384. 21155164

[2] Slocum B, Slocum TD. Tibial plateau levelling osteotomy for repair of cranial cruciate ligament rupture in the canine. Vet Clin North Am Small Anim Pract. 1993; 23(4): 777–795. doi: 10.1016/s0195-5616(93)50082-7 8337790

[3] Montavon PM, Damur DM, Tepic S. Advancement of the tibial tuberosity for the treatment of cranial cruciate deficient stifle. 2002 September 5–8.

[4] Etchepareborde S, Brunel L., Bollen G, M Balligand. Preliminary experience of a modified Maquet technique for repair of cranial cruciate ligament rupture in dogs. Vet Comp Orthop Traumatol. 2011; 24(3): 223–227. doi: 10.3415/VCOT-10-01-0012 21327289

[5] Ness MG. Orthofoam MMP wedge for canine cruciate disease. User guide (version V1. 2). UK, Orthomed. 2014.

[6] Ness MG. The Modified Maquet Procedure (MMP) in Dogs: Technical Development and Initial Clinical Experience. J Am Anim Hosp Assoc. 2016; 52(4): 242–250. doi: 10.5326/JAAHA-MS-6304 27259021

[7] Voss K, Imhof J, Kaestner S, Montavon PM. Force plate gait analysis at the walk and trot in dogs with low-grade hindlimb lameness. Vet Comp Orthop Traumatol. 2007; 20(4): 299–304. doi: 10.1160/vcot-07-01-0008 18038008

[8] Bertram JE, Lee DV, Case HN, Todhunter J. Comparison of the trotting gaits of Labrador Retrievers and Greyhounds. Am J Vet Res. 2000; 61(7): 832–838. doi: 10.2460/ajvr.2000.61.832 10895909

[9] Molsa SH, Hielm-Bjorkman AK, Laitinen-Vapaavuori OM. Force platform analysis in clinically healthy Rottweilers: comparison with Labrador Retrievers. Vet Surg. 2010; 39(6): 701–707. doi: 10.1111/j.1532-950X.2010.00651.x 20345537

[10] Conzemius MG, Evans RB, Besancon F, Gordon WJ, Horstman CL, Hoefle WD, et al. Effect of surgical technique on limb function after surgery for rupture of the CCL in dogs. J Am Vet Med Assoc. 2005; 226(2): 232–236. doi: 10.2460/javma.2005.226.232 15706973

[11] Voss K, Damur DM, Guerrero T, Haessig M, Montavon PM. Force plate gait analysis to assess limb function after tibial tuberosity advancement in dogs with cranial cruciate ligament disease. Vet Comp Orthop Traumatol. 2008; 21(3): 243–249. 18536851

[12] Knebel J, Eberle D, Steigmeier-Raith S, Reese S, Meyer-Lindenberg A. Outcome after Tibial Plateau Levelling Osteotomy and Modified Maquet Procedure in Dogs with Cranial Cruciate Ligament Rupture. Vet Comp Orthop Traumatol. 2020; 33(3): 189–197. doi: 10.1055/s-0040-1701502 32316060

[13] Livet V, Baldinger A, Viguier É, Taroni M, Harel M, Carozzo C, et al. Comparison of Outcomes Associated with Tibial Plateau Levelling Osteotomy and a Modified Technique for Tibial Tuberosity Advancement for the Treatment of Cranial Cruciate Ligament Disease in Dogs: A Randomised Clinical Study. Vet Comp Orthop Traumatol. 2019; 32(4): 314–323. doi: 10.1055/s-0039-1684050 30943550

[14] Amimoto H, Koreeda T, Wada N. Evaluation of recovery of limb function by use of force plate gait analysis after tibial plateau levelling osteotomy for management of dogs with unilateral cranial cruciate ligament rupture. Am J Vet Res. 2019; 80(5): 461–468. doi: 10.2460/ajvr.80.5.461 31034268

[15] Cook JL, Evans R, Conzemius MG, Lascelles DX, McIlwraith W, Pozzi A, et al. Proposed Definitions and Criteria for Reporting Time Frame, Outcome, and Complications For Clinical Orthopedic Studies in Veterinary Medicine. Vet Surg. 2010; 39(8): 905–908. doi: 10.1111/j.1532-950X.2010.00763.x 21133952

[16] Vilar JM, Morales M, Santana A, Spinella G, Rubio M, Cuervo B, et al. Controlled, blinded force platform analysis of the effect of intraarticular injection of autologous adipose-derived mesenchymal stem cells associated to PRGF-Endoret in osteoarthritic dogs. BMC Vet Res. 2013; 9(131): 339–346. doi: 10.1186/1746-6148-9-131 23819757 PMC3716942

[17] Voss K, Galeandro L, Wiestner T, Haessig M, Montavon PM. Relationships of body weight, body size, subject velocity, and vertical ground reaction forces in trotting dogs. Vet Surg. 2010: p. 863–869. doi: 10.1111/j.1532-950X.2010.00729.x 20825596

[18] Vezzoni A, Tavola F. Early diagnosis of canine hip dysplasia (CHD). Veterinaria (Cremona). 2015; 29(6): 7–39.

[19] Portela DA, Verdier N, Otero PE. Review Regional anaesthetic techniques for the pelvic limb and abdominal wall in small animals: A review of the literature and technique description. Vet J. 2018; 238: 27–40. doi: 10.1016/j.tvjl.2018.07.003 30103913

[20] Hong JY, Ko SY, Lee W, Chang YY, Kim SH, Yun JH. Enhancement of Bone Ingrowth into a Porous Titanium Structure to Improve Osseointegration of Dental Implants: A Pilot Study in the Canine Model. Materials. 2020; 13(14): 3061. doi: 10.3390/ma13143061 32650581 PMC7412235

[21] Crovace AM, Staffieri F, Monopoli D, Artiles A, Fracassi L, Crovace A, et al. Role of Tibial Tuberosity Fracture/Fissure through the Maquet Hole in Stifle Osteoarthritis after Porous Tibial Tuberosity Advancement in Dogs at Mid-Term Follow-Up. Vet Sci. 2020; 7(1). 10.3390/vetsci7010001 PMC7157639 31877885

[22] Lefebvre MD, Broux OR, Barthélémy NP, Hamon M, Moyse EV, Bouvy BM, et al. Risk factors for tibial damage associated with the modified Maquet technique in 174 stifles. Vet Surg. 2018; 47(1): 30–35. doi: 10.1111/vsu.12707 29135041

[23] Ramirez J, Barthélémy N, Noël S, Claeys S, Etchepareborde S, Farnir F, et al. Complications and outcome of a new modified Maquet technique for treatment of cranial cruciate ligament rupture in 82 dogs. Vet Comp Orthop Traumatol. 2015; 28(5): 339–346. doi: 10.3415/VCOT-14-10-0153 26219544

[24] Evans R, Gordon W, Conzemius M. Effect of velocity on ground reaction forces in dogs with lameness attributable to tearing of the cranial cruciate ligament. Am J Vet Res. 2003; 64(12): 1479–1481. doi: 10.2460/ajvr.2003.64.1479 14672423

[25] Krotscheck U, Nelson SA, Todhunter RJ, M. Stone ZZ. Long Term Functional Outcome of Tibial Tuberosity Advancement vs Tibial Plateau Leveling Osteotomy and Extracapsular Repair in a Heterogeneous Population of Dogs. Vet Surg. 2016; 45(2): 261–268. doi: 10.1111/vsu.12445 26768085

[26] Voss K, Wiestner T, Galeandro L, Hässig M, Montavon PM. Effect of dog breed and body conformation on vertical ground reaction forces, impulses, and stance times. Vet Comp Orthop Traumatol. 2011: p. 106–112. doi: 10.3415/VCOT-10-06-0098 21243175

[27] Volstad NJ SG, RS BSC. The evaluation of limb symmetry indices using ground reaction forces collected with one or two force plates in healthy dogs. Vet Comp Orthop Traumatol. 2017; 30: 54–58. doi: 10.3415/VCOT-16-04-0054 27849103

